# Supplementary material for: The genome sequence of the Osiris Blue, Cupido osiris (Meigen, 1830) (Lepidoptera: Lycaenidae)
Source: Wellcome Open Res. 2026 Jan 23;11:71. [Version 1] doi: 10.12688/wellcomeopenres.25821.1 (PMC12963831; doi:10.12688/wellcomeopenres.25821.1)
Supplement: Peer review history

# Reviewer report: Reviewer response for version 1

**DOI:** 10.21956/wellcomeopenres.28422.r146650

**Published:** 2026-03-05

**Authors:** Sivasankaran Kuppusamy

**Affiliations:** 1 Loyola College, chennai, Tamil Nadu, India

The study reports a high-quality chromosome-level genome sequence of *Cupido osiris* (Meigen, 1830). The assembly consists of two haplotypes with lengths of 481.43 Mb and 479.83 Mb, respectively. This research provides important molecular data for Lepidopteran studies and contributes valuable resources for evolutionary, comparative, and conservation genomics.

 The use of PacBio HiFi sequencing combined with Hi-C scaffolding ensures high accuracy and strong structural integrity of the assembly. The bioinformatics pipeline appears standard, reproducible, and well-documented.

 The application of advanced sequencing and assembly methods is clearly demonstrated by the successful separation into two haplotypes, which increases precision by distinguishing maternal and paternal chromosome sets.

 Approximately 98.72% of haplotype 1 was scaffolded into 24 chromosomal pseudomolecules, indicating near chromosome-level completeness. Genome completeness assessment using BUSCO demonstrates high contiguity, strong completeness, and excellent base-level accuracy.

 Comments on the Manuscript

 A total of 207.89 Gb of sequencing data were generated from two different platforms. However, following assembly and annotation, the manuscript does not report the number of predicted protein-coding genes, non-coding genes, or gene transcripts. These important annotation statistics should be clearly presented in the main text and summarized in a table for clarity.

 Overall Comments

 The manuscript is well prepared and suitable for scientific indexing.

Are sufficient details of methods and materials provided to allow replication by others?

Yes

Is the rationale for creating the dataset(s) clearly described?

Yes

Are the datasets clearly presented in a useable and accessible format?

Yes

Are the protocols appropriate and is the work technically sound?

Yes

Reviewer Expertise:

Phylogenetic analysis of superfamily Noctuoidea moths using mitogenome sequences

I confirm that I have read this submission and believe that I have an appropriate level of expertise to confirm that it is of an acceptable scientific standard.

# Reviewer report: Reviewer response for version 1

**DOI:** 10.21956/wellcomeopenres.28422.r146031

**Published:** 2026-02-19

**Authors:** Sophie Tandonnet, María Morán Saiz

**Affiliations:** 1 Universitat de Barcelona, Barcelona, Spain

In this genome note, the authors report the chromosome-scale assembly for the genome of Osiris Blue butter fly ( *Cupido osiris*). The assembly originates from a single male (the homogametic sex) was assembled in two haplotypes. Haplotype 1 was curated to chromosome level and contains 24 pseudo-chromosomal molecules (including the Z chromosome). This chromosome number differs from the usual 31 chromosomes found in Lepidoptera. Looking at the Merian element pattern, some recent fusions are quite clear. This haplotype is of high quality (QV 59.3), completeness (97.4% complete BUSCOs) and highly continuous (98.72% of the genome in chromosome-scale scaffolds). The assembly is based on Pacbio HiFi long reads (thorax used) and scaffolded into pseudo-chromosomal molecules using Hi-C data (head and abdomen used). The same individual (ilCupOsir1) was used for the Pacbio HiFi sequencing and the Hi-C. The assembly doesn't have an annotation yet. The paper is straightforward and easy to follow.

 All in all, this note reports a new, high-quality and high-continuity genome assembly of a species not yet sequenced.

**Main Comments**

- It is unfortunate that the homogametic sex was sequenced instead of the heterogametic sex. The W chromosome is therefore missing and no coverage analysis can be performed to identify the Z chromosome. This severely limits studies focusing on sex determination/differentiation, sex chromosome evolution, general comparative genomics analyses and studies aiming to understand the sexual dimorphism in this species. Although Lepidoptera’s macrosynteny is very conserved (we particularly like the inclusion of the Merian element painting), coverage analysis to uncover the Z chromosome should be preferred and used in conjunction with homology techniques to identify sex chromosomes. Just homology cannot identify sex chromosomes that have newly evolved (appearance of neo-W and neo-Z). 
- No annotation or RNA-seq data is available for this species which also limits its usage as a resource. Nevertheless, this could be done at a later stage.
- The link to the data is correct and the data available for download.
- The background on *Cupido osiris* was nice to read.
- The picture of the specimen is lacking of the parts that were used for the sequencing. 

Are sufficient details of methods and materials provided to allow replication by others?

Yes

Is the rationale for creating the dataset(s) clearly described?

Yes

Are the datasets clearly presented in a useable and accessible format?

Yes

Are the protocols appropriate and is the work technically sound?

Yes

Reviewer Expertise:

Evolutionary Biology, sex determination, genetics, genomics

We confirm that we have read this submission and believe that we have an appropriate level of expertise to confirm that it is of an acceptable scientific standard.
